# High eosinophil blood counts are associated with a shorter length of hospital stay in exacerbated COPD patients – a retrospective analysis

**DOI:** 10.1186/s12931-020-01365-5

**Published:** 2020-05-06

**Authors:** Timm Greulich, Julia Tüffers, Sina Mager, Anna Eder, Michael Maxheim, Peter Alter, Bernd Schmeck, Claus F. Vogelmeier

**Affiliations:** 1 Department of Medicine, Pulmonary and Critical Care Medicine, University Medical Centre Giessen and Marburg, Philipps-University, Centre for Lung Research (DZL), 35043 Marburg, Germany; 2 Department of Pneumology and Critical Care Medicine, Cologne-Merheim Hospital, ARDS and ECMO Centre, Kliniken der Stadt Köln gGmbH, Witten/Herdecke University Hospital, 51109 Cologne, Germany; 3 Institute for Lung Research, Philipps-University, Centre for Lung Research (DZL), 35043 Marburg, Germany

**Keywords:** COPD, Eosinophil, Infection and inflammation

## Abstract

**Background:**

In COPD, the course of the disease including morbidity and mortality is strongly associated with severe exacerbations. The current GOLD recommendations emphasize blood eosinophil counts as a marker for responsiveness to inhaled corticosteroids (ICS). Retrospective analyses from randomized clinical trials indicate a favorable response to systemic corticosteroids in exacerbated COPD patients with blood eosinophils > 2%, however data outside clinical trials are scarce.

**Patients and methods:**

We retrospectively evaluated data from 1007 cases of patients who were admitted to the University Medical Center Marburg between 01/2013 and 12/2018. All patients had been diagnosed with an acute exacerbation of COPD (ICD-10 J44.0/J44.1). Our analysis was based on a subgroup of 417 patients in whom a full blood cell count was obtained at the day of admission. Patients were predominantly male (63.3%), had a median age of 74 years (IQR 65 years – 83 years) and a median FEV1 of 1.03 l (42.6% predicted). We compared the hospital length of stay and other outcome parameters using established thresholds for the eosinophil blood cell count (100 and 300 eosinophils/μl and 2%).

**Results:**

Patients with low eosinophils (< 2%, <100 cells/μl) had a longer median time in hospital (length of hospital stay – LOS) as compared to patients with high eosinophils (< 2%: 9.31 vs. ≥2%:7 days, and < 100/μl: 10 vs. 100–300/μl: 8 vs. > 300/μl: 7 days). The median CRP was higher in patients with low eosinophils as compared to the other groups (< 2%: 22.7 vs. ≥2%: 9 mg/dl and < 100: 25 vs. 100–300: 13.5 vs. > 300: 7.1 mg/dl). Time to re-hospitalization or time to death did not differ between strata of eosinophils. Sensitivity analysis in a subgroup of patients in which pneumonia was excluded by chest x-ray did not significantly alter the results.

**Conclusion:**

The results support the hypothesis that patients with severe COPD exacerbations and elevated blood eosinophil counts respond better to systemic corticosteroid treatment than patients with a non-eosinophilic exacerbation.

## Background

Morbidity and mortality of Chronic Obstructive Pulmonary Disease (COPD) is strongly associated with severe exacerbations [1].

The current GOLD recommendations emphasize blood eosinophils (eos) as a marker for responsiveness to inhaled corticosteroids (ICS) [2]. The recommended thresholds are < 100 cells/μl and > 300 cells/μl. These recommendations are based on a high number of analyses from both, population based cohort studies and secondary analyses from randomized clinical trials: In a subgroup of COPD patients of the Copenhagen General Population Study, higher baseline blood eos were associated with an increased incidence for severe exacerbations [3]. Similar findings have also been recognized in a number of randomized controlled trials comparing treatment with LABA as compared to ICS/LABA [4, 5]. In a post-hoc-analysis of the WISDOM-study, Watz et al. found that higher blood eosinophils were associated with an increased exacerbation rate after steroid withdrawal in patients pre-treated with triple therapy [6]. Further evidence comes from pharmaceutical parallel-groups randomized clinical trials in which inhaled triple therapy has been compared to different dual combinations. Higher blood eosinophils were usually associated with higher ICS efficacy [7–9].

The therapeutic and prognostic implications of blood eosinophils during an exacerbation are less clear: In a post-hoc analysis Bafadhel et al. evaluated the rate of recovery of eosinophilic (blood eos ≥200 cells/μl and/or ≥ 2%) and non-eosinophilic exacerbations from COPD subjects (*n* = 243) who participated in a multi-centre randomised control trial [10]. In the eosinophilic exacerbation population, the length of hospital stay was shorter than in patients with non-eosinophilic exacerbations following treatment with oral corticosteroids. The CRP tended to be normal [10].

Because data from real life settings confirming these RCT-based findings are scarce, we retrospectively evaluated the association between baseline eosinophil counts and clinical outcomes in patients hospitalized due to an exacerbation of their underlying COPD.

## Methods

### Patient population

In this analysis, we present retrospectively collected data from 1007 exacerbations which were treated at the University Medical Center Marburg between 01/2013 and 12/2018. All patients had been diagnosed with an acute exacerbation of COPD according to the ICD code (J44.0/J44.1). According to national guidelines valid at the time of data collection, there were no standardized criteria for hospital admission due to acute exacerbation. The decision was based on the patients’ clinical impression, reflected by the metabolic status (assessed by blood gas analysis), breathing frequency and oxygen saturation.

Patients were predominantly male (65%), had a median age of 72 years (IQR 64 years – 81 years) and a median FEV_1_ of 1.02 l (39.3% predicted). Our analysis was based on a subgroup of 417 cases of patients in whom a full blood cell count was obtained at the day of admission. These patients exhibited similar baseline characteristics (63,3% male, 74 years of age, FEV_1_ 1.03 l, 42.6% predicted) (Table 1).

**Table 1:** Baseline characteristics

	All	< 100 [eos/μl]	100–300 [eos/μl]	> 300 [eos/μl]	< 2 [%]	≥2 [%]
N	417	219	129	69	276	141
Age [years]	74.0 (65.0–83.0)	73.0 (63.0–83.0)	73.0 (65.0–81.0)	76.0 (67.0–83.0)	72.0 (64.0–82.0)	76.0 (67.0–83.0)
Male sex [%]	63.3	60.3	67.4.	65.2	61.2	67.4
Packyears	40.0 (30.0–59.0)	40.0 (22.0–50.0)	40.0 (30.0–50.0)	45.0 (30.0–60.0)	40.0 (25.0–50.0)	40.0 (30.0–60.0)
FEV1[l]	1.03 (0.74–1.44)	0.98 (0.71–1.30)	1.06 (0.74–1.50)	1.10 (0.81–1.54)	1.00 (0.71–1.33)	1.11 (0.78–1.50)
FEV1 [pred. %]	42.6 (30.6–54.8)	38.3 (29.5–51.9)	45.3 (30.8–58.2)	42.6 (34.4–58.8)	41.8 (29.1–53.0)	42.6 (33.9–57.6)
FEV1/VC	46.0 (37.1–57.2)	45.2 (35.6–54.5)	44.4 (36.4–62.3)	50.7 (41.4–55.5)	44.8 (35.5–56.0)	47.5 (38.5–58.5)

Displays the baseline characteristics depending on the blood eosinophils on the day of admission. Continuous parameters are expressed as Median and (IQR). Mann-Whitney test was used for two groups comparisons, Kruskal-Wallis test with Dunn’s test for three groups comparisons. No significant differences were detected between the groups.

Chart reviews were done to describe further characteristics of these patients and to evaluate their clinical outcome. From the routine medical records we transferred and analyzed the following parameters: Case Mix Index (CMI) - a formula of the Diagnosis Related Groups (DRG) system for the calculation of patients’ case severity, which is used as a controlling instrument of hospitals relevant for financial reimbursement - as a rough marker of disease severity, inflammatory markers, other basic laboratory values, and spirometry. As primary endpoint, we compared the length of hospital stay. Further analyses described other markers of disease severity and outcome (re-hospitalization and mortality within our hospital as well as the annual severe exacerbation rate within our hospital) according to the above mentioned eosinophil blood cell count strata (100 and 300 eosinophils/μl and 2% [2, 10–12]).

As this was a purely retrospective analysis from clinical routine data, we obtained a waiver by the ethics committee because a formal review was considered as not mandatory (EM_MR_greulich_130320).

### Subgroup analyses

Two separated subgroup analyses were performed: Firstly, we analyzed a subgroup of 243 cases of patients, who – according to the medical records - did not receive systemic steroids before the full blood count was obtained. Baseline characteristics of these patients were similar to the primary analysis population and can be seen in supplementary Table S1.

Secondly: For the vast majority of cases admitted to our hospital, chest x-rays were performed as part of clinical routine (> 95%). As exacerbations of COPD and pneumonia may overlap in some cases, we performed an additional analysis, excluding patients with radiological signs of pneumonia (*n* = 89), leaving a study population of 322 exacerbations. Baseline characteristics were similar and can be seen in supplementary Table S2.

### Statistical analyses

As data were not normally distributed, Mann-Whitney tests and Kruskal-Wallis tests were applied to compare metric outcome variables between different eosinophil strata. The exact fisher test was applied to compare categorical variables. For patients that were re-hospitalized in our hospital or died in our hospital during the observational time period, Kaplan-Meier curves were used to visualize the results and Wilcoxon log rank test was applied to compare the data between the strata. IBM SPSS statistics version 24 (IBM, Armonk, New York, USA) was used for all calculation, graphs were constructed using IBM SPSS or GraphPad version 7 (GraphPad, San Diego, CA, USA).

## Results

Of the 417 cases of patients that were included in the study, 276 (66.2%) had a peripheral blood eosinophil count of < 2%, whereas 141 cases (33.8%) had ≥2% eosinophils of the total leucocyte count. In the absolute strata, there were 219 exacerbations (52.5%) with eosinophil counts < 100 cells/μl, 129 (30.9%) with eosinophils between 100 and 300 cells/μl and 69 (16.5%) that exhibited absolute eosinophilic cell counts > 300/μl.

### Length of hospital stay

**Fig. 1:**
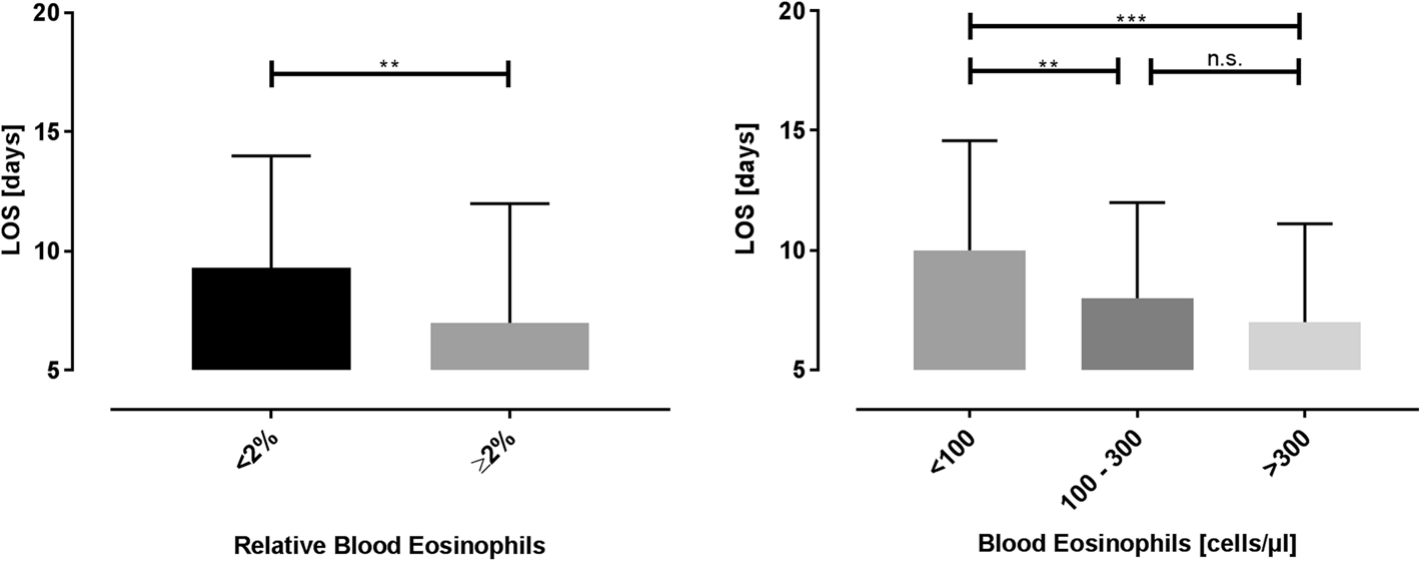
Hospital length of stay according to the blood eosinophils at the day of admission. Displays the median (+ IQR) hospital length of stay according to the blood eosinophils at the day of admission. Patients with a low relative eosinophil count experienced a longer hospital stay as compared to patients with a high relative eosinophil count (left diagram; *p* < 0,005; Mann-Whitney test). Patients with low absolute number of blood eosinophils also exhibited a longer length of hospital stay as compared to patients with an intermediate or high number of blood eos (right diagram; *p* < 0.005; *p* < 0.001; Kruskal-Wallis test with Dunn’s test)

Patients with low eosinophils (< 2%, <100 cells/μl) had a longer median time in hospital (length of hospital stay – LOS) as compared to patients with high eosinophils (Fig. 1). This was true for both, relative and absolute values.

Restricting the analysis to patients naïve for systemic steroids (before blood collection) yielded similar outcomes: The median LOS in patients with low eosinophils was longer than in their respective control group (< 2%: 9 vs. ≥ 2%: 7 days, *p* < 0.05). The same applied to the absolute eosinophilic groups (< 100/μl: 9.18 vs. 100–300/μl: 7 days, n.s.; 100–300/μl: 7 vs. > 300/μl: 6.54 days, n.s.; < 100/μl: 9.18 vs. > 300/μl: 6.54 days, *p* < 0.05).

Additionally, restricting the analysis to patients without consolidation on chest x-ray yielded similar results, too: The median LOS in patients with low eosinophils was longer than in patients with a higher relative number of blood eosinophils (< 2%: 9 vs. ≥ 2%: 7 days, *p* < 0.05). Similar trends could be observed regarding strata derived from absolute numbers of eosinophils (< 100/μl: 9.96 vs. 100–300/μl: 8 n.s.; 100–300/μl: 8 vs. > 300/μl: 6.54 days, n.s.; < 100/μl:9.96 vs. > 300/μl: 6.54 days, *p* < 0.01).

### Inflammatory markers

**Fig. 2:**
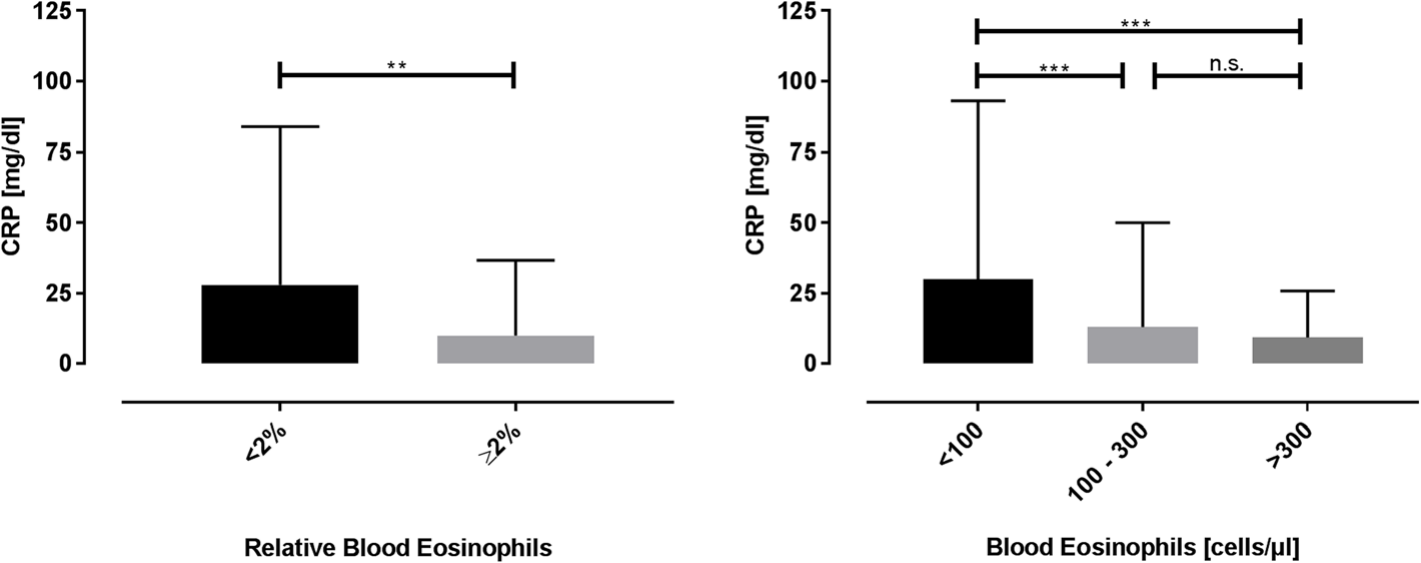
C-reactive protein according to the blood eosinophils at the day of admission. Shows the median (+ IQR) C-reactive protein (CRP) according to the blood eosinophils at the day of admission. Patients with a low relative eosinophil count showed a higher CRP than patients with a high relative eosinophil count (left diagram; *p* < 0.001; Mann-Whitney test). Patients with low absolute numbers of blood eosinophils also exhibited a higher CRP than patients with an intermediate or high number of blood eos (right diagram; *p* < 0.001; *p* < 0.001; Kruskal-Wallis test with Dunn’s test)

The median CRP was higher in patients with low eosinophils as compared to the other groups (Fig. 2). The same trend could be observed for a number of further inflammatory markers (Table 2).

**Table 2:** Inflammatory markers

	All	< 100 eos/μl	100–300 eos/μl	> 300 eos/μl	***P***-value	< 2%	≥2%	***P***-value
**N**	417	219	129	69		276	141	
**Leucocytes [g/l]**	11,2 (8,32 - 14,8)	12,0 (8,41 -16,5)	11,2 (8,49 - 13,5)	9,98 (7,99 -14,6)	n.s.	12,5 (9,06 -16,1)	9,38 (7,67 - 12,2)	§§§
**Neutrophils [%]**	78,0 (68,0 - 88,0)	85,0 (76,0 - 91,0)	73,0 (65,0 - 81,0)	65,0 (56,0 - 76,5)	*** ### $$	83,5 (76,0 - 90,0)	67,0 (57,5 - 76,0)	§§§
**Procalcitonin [μg/l]**	0,05 (0,05 - 0,20)	0,10 (0,05 - 0,33)	0,05 (0,05 - 0,20)	0,05 (0,05 - 0,05)	##	0,10 (0,05 - 0,25)	0,05 (0,05 - 0,10)	§
**Fibrinogen [g/l]**	5,05 (4,00 - 6,80)	5,30 (4,10 -14,0)	5,00 (3,80 - 6,43)	4,50 (3,50 - 5,80)	##	5,14 (4,00 - 14,0)	4,70 (3,65 - 6,08)	§
**CRP [mg/l]**	20,0 (6,00–66,5)	30,0 (12,0 - 93,0)	13,0 (2,50–50,0)	9,25 (2,50 - 25,8)	*** ###	28,0 (10,0 - 83,9)	10,0 (2,50 - 36,6)	§§§

Median and IQR of inflammatory markers according to the blood eosinophils at the day of admission. In general, patients with a low relative eosinophil count showed a higher inflammatory markers as compared to patients with higher eosinophils (Mann-Whitney test for two groups comparisons, Kruskal-Wallis test with Dunn’s test for three groups comparisons). *N* = 417 for leukocytes and granulocytes, *n* = 156 for Procalcitonin, *n* = 406 for fibrinogen; * significant between < 100 and 100–300; # significant between < 100 and > 300; $ significant between 100 and 300 and > 300; § significant between < 2% and ≥ 2%

Subgroup analyses regarding inflammatory markers (firstly, excluding cases of exacerbations in non-systemic-steroid-naïve patients; secondly ruling out cases with signs of pneumonia in the chest x-ray) confirmed our analyses in trends at similar significant levels (supplementary Table S3 and Table S4).

### Case mix index

A significant difference could be established between the Case Mix Index in the different strata of eosinophil blood counts. The analysis showed that patients with a lower relative eosinophilic count recorded a higher median CMI than those with a relative eosinophilic count ≥2% (< 2%: 0,91 vs. ≥2%: 0,74, *p* < 0,0001). The same applied to the absolute eosinophilic counts and the particular thresholds (<100cells/μl: 0,91 vs. 100-300cells/μl: 0,74 vs. > 300cells/μl: 0,74, *p* < 0,01).

### Longitudinal analyses

**Fig. 3:**
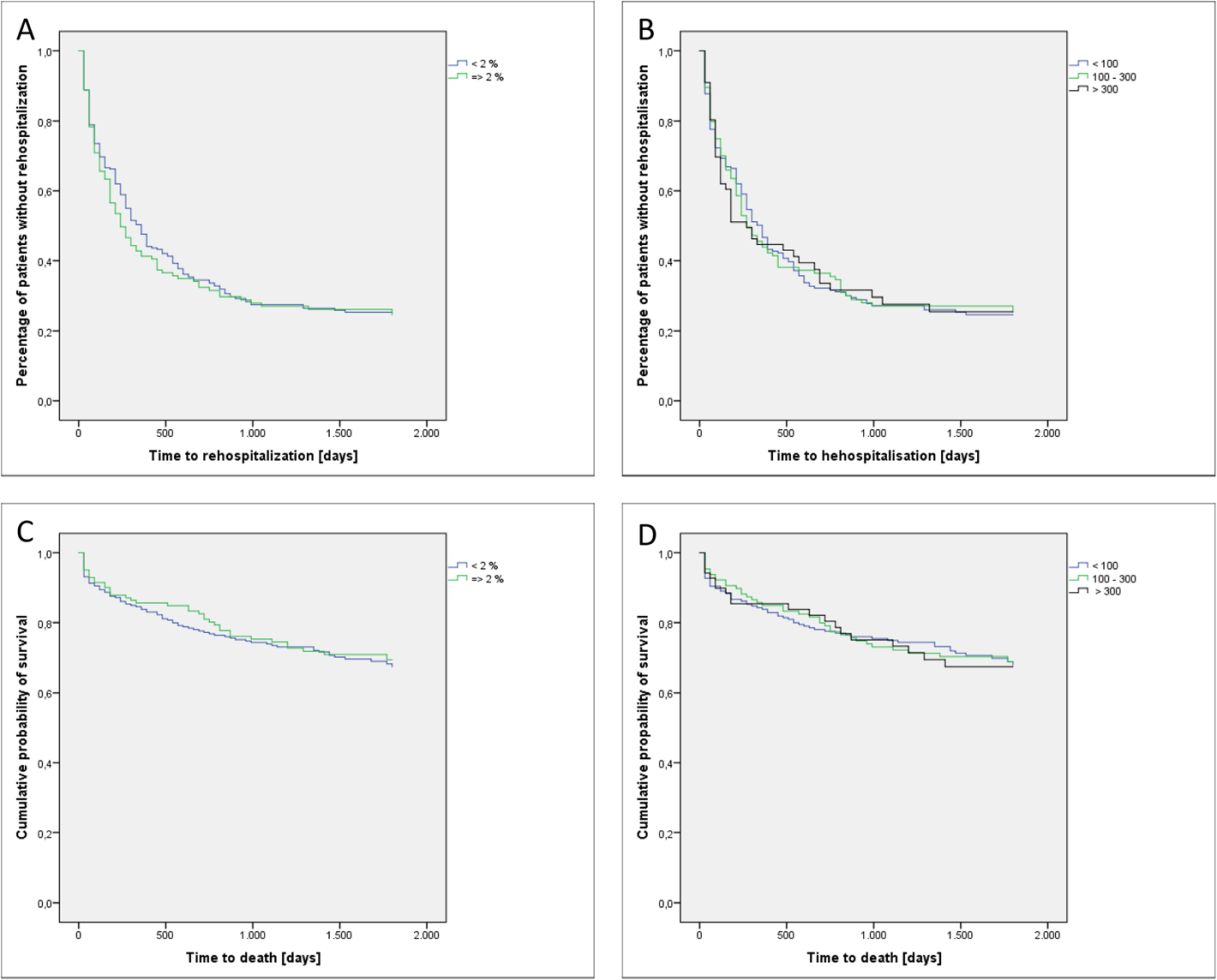
Survival rates according to the blood eosinophils at the day of admission. Time to re-hospitalization (**a**) and **b**)) or death (**c**) and **d**)) according to the blood eosinophils at the day of admission. No significant differences were detected (Log-Rank Mantel-Cox) between the groups

Overall 72.66% of the 417 included exacerbated patients experienced a re-hospitalization to our hospital (median follow-up of 254 days) while 28.78% died during follow-up in our hospital (median follow-up of 1469 days). Time to hospital readmission (Fig. 3 a and b) or time to death (in-hospital-mortality only, Fig. 3c and d) did not differ between the groups. Repeating the analysis in the above defined subgroups did not alter the results (data not shown).

Of all 417 cases of patients with a full cell blood count at the day of admission, we observed the annual severe exacerbation rate until the date of death or endpoint of this study (31.12.2018) according to the levels of blood eosinophil counts. In both relative and absolute strata, the differences in median exacerbation rate were not statistically significant (< 2%: 0.27 vs. ≥2%: 0.25 annual exacerbations in the relative and < 100/μl: 0.27 vs. 100–300/μl: 0.26 vs. > 300/μl: 0.29 in the absolute strata).

## Discussion

In our retrospective analyses, patients suffering from a non-eosinophilic exacerbation (< 2% or < 100 cells/μl) stayed in the hospital for a longer period of time as compared to patients exhibiting a higher eosinophil count at the day of admission. These patients tended to demonstrate a stronger general inflammatory reaction, based on their levels and numbers of CRP, leucocytes, neutrophile fraction, PCT, and fibrinogen. The nature of the exacerbation was not relevant with regard to re-hospitalization (due to COPD) and/or death (in our hospital). Our real world data confirm and enhance existing data from clinical trials and other (mainly smaller) cohorts.

Comparing our study to published results from other studies, at least three important manuscripts have to be taken into account. Bafadhel et al. analyzed 243 COPD subjects at presentation to hospital with an exacerbation that participated in a multi-center randomized controlled trial in Great Britain evaluating early rehabilitation [10]. Duman et al. performed a retrospective chart review in a tertiary teaching hospital, recruiting 1704 patients hospitalized with COPD exacerbation [13] in a large hospital in Turkey. MacDonald et al. conducted two cohort studies (*n* = 242 for the restrospectively collected derivation cohort, *n* = 99 for the prospectively collected validation cohort) observing patients hospitalized for an acute exacerbation of COPD in Melbourne [14].

In all four studies, the percentage of eosinophilic exacerbations was roughly comparable. It was approximately 20% (threshold: ≥2%) in the British and Turkish studies and 33,8% in our study. The same was true if absolute eosinophils were used to stratify the groups (> 300 cells/μl defined as eosinophilic): We found 16,3% to be eosinophilic while the colleagues in Melbourne found 25,2%. Taken together, one quarter to one third of exacerbations seem to be eosinophilic regardless of the setting or continent in which the studies have been performed.

Regarding outcome parameters, again we find our results in line with the other observations: Although assessed with different methods (comparison of means, comparison of median values, Kaplan-Meyer analysis) in every study, the length of hospital stay was significantly longer in patients with low eosinophils as compared to patients experiencing an eosinophilic exacerbation. Bafadhel et al. described different clusters of exacerbations, leading to different exacerbation phenotypes [15]. This finding provides a logical explanation for the differential responsiveness to ICS-treatment of COPD patients, dependent on the number blood eosinophils [4, 16, 17]. One may speculate that the association between acute severe exacerbations with eosinophilic inflammation and a shorter length of hospital stay may be due to a rapid response to steroid treatment. An alternative explanation would be that that differences in the type of ICS prescribed could be responsible for the differences in treatment response. However, analyzing the distribution of different ICS in the eosinophilic and non-eosinophilic strata, yielded no significant differences (supplementary Table S5).

Regarding parameters of inflammation we present similar results to the studies of Bafadhel, MacDonald and Duman [10, 13, 14]. We confirm their findings that lower eosinophils are associated with higher levels of inflammation. As the median procalcitonin was higher in this group, this might reflect more bacterial inflammation [18, 19]. We enhance the number of parameters by demonstrating that this is not only true for CRP but also for PCT, leukocytes, neutrophils, and fibrinogen. Taken together with the longer hospital stay these findings again support the existence of different exacerbation phenotypes and the notion that a rather non-eosinophil phenotype (which may include bacterial or viral infections) may need a longer time to clinical improvement.

With regard to the sometimes challenging differential diagnosis between a bacterial exacerbation of COPD and a community-acquired pneumonia, we tried to account for that by repeating our analysis after having excluded patients with signs of consolidation on chest x-ray, which did not change our results. In our opinion, this further reinforces the generalizability of the results.

A high CMI was associated with low blood eosinophil counts, whereas the opposite applied to patients with high eosinophils. These findings go along with the previously discussed results (longer LOS and higher level of inflammation in patients with low eosinophil blood counts) and support our hypothesis of a more rapid response to corticosteroid treatment in patients with eosinophilic exacerbations. However, the possibility has to be taken into account that a high disease severity itself, reflected by a high CMI, might also be the reason for the strongly varying courses of disease in patients with acute exacerbations of COPD. Due to the retrospective nature of the study we do not see a way to further examine this matter.

With regard to long-term outcome, we did not find any correlation between exacerbation phenotype and re-hospitalization or death. This is in line with the results of Bafadhel and Duma [10, 13] but contradicts what has been found by MacDonald and colleagues [14]. A number of explanations are possible: As our study is based on chart reviews only and no formal assessment of survival has been accomplished, we can only report re-hospitalization and/or death that occurred in our hospital. Furthermore, long term health outcomes in COPD may be more closely related to disease severity [20], number of exacerbations [21], or comorbidities [22, 23].

A relatively new finding of the current study is that lung function was worse in patients with a non-eosinophilic exacerbation (significant only in subgroup analysis. In two prospective clinical studies, Crisafulli et al. analyzed clinical predictors of a) treatment failure within 7 days and b) prolonged hospital stay in patients with severe exacerbations of COPD. In the first study they found that treatment failure was associated with worse lung function, while in the second study there was no difference in lung function between patients with a hospital stay ≤7 and > 7 days [24, 25]. Furthermore, in a recently published retrospective study, Tang et al. investigated the relationship of blood eosinophilia with pulmonary function parameters in 247 exacerbated COPD patients [26]. They also found that patients with high levels of blood eosinophil counts (≥2% eos) had better lung function than patients in the non-eosinophlic group (< 2% eos). This goes in line with a prospective observational study of Ko et al., who detected an improvement of FEV1 in 346 exacerbated COPD patients with eosinophil blood counts ≥2% after 12 months, when compared to the spirometry records at baseline [27]. Since we did not differentiate whether spirometry was performed in the beginning of admission or towards the end of the hospital stay (after treatment response), we cannot systematically assess whether lung function was a confounding factor or a treatment result. As our data are in line with other studies, we think both explanations may be possible.

There are a number of limitations to our study: Firstly, the retrospective study design limits the interpretation of our data. Secondly, no standardized discharge criteria have been used. Thirdly, we could not assess re-hospitalization or mortality that occurred outside our hospital. Fourthly, although recommended by guidelines and hospital standard operating procedures, in some cases systemic steroids may not have been administered. However, repeating the analysis without cases in which systemic steroid treatment had not been documented did not change the results significantly (data not shown). Fifthly, we did not assess the exacerbation phenotype systematically. Albeit these limitations, the present study adds to the growing body of evidence that blood eosinophils may serve as a biomarker not only for ICS-responsiveness with regard to the prevention of exacerbations but also for responsiveness towards systemic steroids during an acute exacerbation of COPD. We enhance the generalizability of pre-existing data by introducing real-world data (as compared to RCT data [10]) from a high-standard Western European country (as compared to the other available studies [13, 14]).

## Conclusion

In summary, our data support the hypothesis that patients with low eosinophil counts may be less responsive to systemic corticosteroids when compared to patients with high eosinophils. This may translate into a longer hospital length of stay.

## Supplementary information

**Additional file 1: Table S1** displays the baseline characteristics according to the blood eosinophils of patients in whom systemic steroids were administered only after the blood samples had been taken. Continuous parameters are displayed as Median and (IQR). Chi-Square test was performed to compare gender between the groups. For continuous parameters: Mann-Whitney test was calculated for two groups comparisons, Kruskal-Wallis test with Dunn’s test for three groups comparisons. * significant between < 100 and 100–300; # significant between < 100 and > 300; $ significant between 100 and 300 and > 300; § significant between < 2% and ≥ 2%. **Table S2** displays the baseline characteristics of patients without any signs of pneumonia (*n* = 322). Continuous parameters are displayed as Median and (IQR). Chi-Square test was performed to compare gender between the groups (& indicates a significantly different gender distribution). For continuous parameters: Mann-Whitney test was calculated for two groups comparisons, Kruskal-Wallis test with Dunn’s test for three groups comparisons. * significant between < 100 and 100–300; # significant between < 100 and > 300; $ significant between 100 and 300 and > 300; § significant between < 2% and ≥ 2%. **Table S3** displays inflammatory markers according to the blood eosinophils of patients in whom systemic steroids were administered only after the blood samples had been taken. Continuous parameters are displayed as Median and (IQR). *N* = 243 for leukocytes, *n* = 238 for neutrophils, *n* = 86 for Procalcitonin, *n* = 234 for fibrinogen. *n* = 239 for CRP; * significant between < 100 and 100–300; # significant between < 100 and > 300; $ significant between 100 and 300 and > 300; § significant between < 2% and ≥ 2%. **Table S4** displays the subgroup analysis of inflammatory markers according to the blood eosinophils in cases of patients without any signs of pneumonia (*n* = 322). Continuous parameters are displayed as Median and (IQR). * significant between < 100 and 100–300; # significant between < 100 and > 300; $ significant between 100 and 300 and > 300; § significant between < 2% and ≥ 2%. **Table S5** displays the distribution of different types of ICS prescribed according to the eosinophil and non-eosinophil thresholds in cases of patients with a differential cell blood count at the day of admission (*n* = 417). Statistical analysis was performed using Chi-square test. Types of ICS that were administered in less than five cases were excluded from this analysis (Ciclesonide and Fluticasone furoate).

## Data Availability

The datasets generated and/or analyzed during the current study are not publicly available due to further planned analyses from the same dataset.

## Abbreviations

COPD: Chronic Obstructive Pulmonary Disease
CMI: Case Mix Index
CRP: C-reactive protein
eos: eosinophils
FEV1: Forced expiratory volume in one second
GOLD: Global Initiative for Chronic Obstructive Lung Disease
ICS: Inhaled corticosteroids
IQR: Interquartile range
LOS: Length of hospital stay
PCT: Procalcitonin
RCT: Randomized controlled trial
VC: Vital capacity
