# Supplementary material for: High eosinophil blood counts are associated with a shorter length of hospital stay in exacerbated COPD patients – a retrospective analysis
Source: Respir Res. 2020 May 6;21:106. doi: 10.1186/s12931-020-01365-5 (PMC7204070; doi:10.1186/s12931-020-01365-5)
Supplement: Supplementary file 1 — Additional file 1: Table S1 displays the baseline characteristics according to the blood eosinophils of patients in whom systemic steroids were administered only after the blood samples had been taken. Continuous parameters are displayed as Median and (IQR). Chi-Square test was performed to compare gender between the groups. For continuous parameters: Mann-Whitney test was calculated for two groups comparisons, Kruskal-Wallis test with Dunn’s test for three groups comparisons. * significant between < 100 and 100–300; # significant between < 100 and > 300; $ significant between 100 and 300 and > 300; § significant between < 2% and ≥ 2%. Table S2 displays the baseline characteristics of patients without any signs of pneumonia (n = 322). Continuous parameters are displayed as Median and (IQR). Chi-Square test was performed to compare gender between the groups (& indicates a significantly different gender distribution). For continuous parameters: Mann-Whitney test was calculated for two groups comparisons, Kruskal-Wallis test with Dunn’s test for three groups comparisons. * significant between < 100 and 100–300; # significant between < 100 and > 300; $ significant between 100 and 300 and > 300; § significant between < 2% and ≥ 2%. Table S3 displays inflammatory markers according to the blood eosinophils of patients in whom systemic steroids were administered only after the blood samples had been taken. Continuous parameters are displayed as Median and (IQR). N = 243 for leukocytes, n = 238 for neutrophils, n = 86 for Procalcitonin, n = 234 for fibrinogen. n = 239 for CRP; * significant between < 100 and 100–300; # significant between < 100 and > 300; $ significant between 100 and 300 and > 300; § significant between < 2% and ≥ 2%. Table S4 displays the subgroup analysis of inflammatory markers according to the blood eosinophils in cases of patients without any signs of pneumonia (n = 322). Continuous parameters are displayed as Median and (IQR). * significant between < [file 12931_2020_1365_MOESM1_ESM.docx]

|  | **All** |  | **<100 [eos/µl]** | **100-300 [eos/µl]** | **>300 [eos/µl]** | **p-value** |  | **<2 [%]** | ≥**2 [%]** | **p-value** |
| --- | --- | --- | --- | --- | --- | --- | --- | --- | --- | --- |
| N | 243 |  | 114 | 76 | 53 |  |  | 148 | 95 |  |
| Age | 73.0  (65.0 - 82.0) |  | 72.0  (62.0 - 82.0) | 72.5  (66.3 - 81.0) | 76.0  (67.5 - 83.0) | n.s. |  | 71.0  (64.0 - 80.0) | 78.0  (68.0 - 83.0) | ^§^ |
| Male sex [%] | 65.4 |  | 64.9 | 64.5 | 67.9 | n.s. |  | 64.9 | 66.3 | n.s. |
| Packyears | 40.0  (30.0 - 60.0) |  | 40.0  (25.0 - 50.0) | 40.0  (30.0 - 57.5) | 45.0  (30.0 - 60.0) | n.s. |  | 40.0  (28.8 - 60.0) | 40.0  (30.0 - 60.0) | n.s. |
| FEV1[l] | 1.05  (0.73 - 1.54) |  | 0.97  (0.70 - 1.30) | 1.05  (0.69 - 1.57) | 1.26  (0.89 - 1.69) | *  ^#^ |  | 0.98  (0.68 - 1.31) | 1.25  (0.79 - 1.69) | ^§^ |
| FEV1 [%pred.] | 42.5  (30.1 - 56.0) |  | 34.4  (24.9 - 51.8) | 45.2  (32.4 - 57.2) | 45.8  (34.8 - 64.4) | ^##^ |  | 36.8  (24.7 - 52.7) | 46.0  (35.2 - 62.0) | ^§§^ |
| FEV1/VC [%] | 45.4  (36.7 - 55.4) |  | 43.7  (35.2 - 49.7) | 43.7  (36.0 - 56.8) | 50.7  (41.2 - 55.9) | n.s. |  | 43.0  (35.1 - 50.1) | 47.5  (38.5 - 56.8) | n.s. |

**Supplement Table 1 displays the baseline characteristics according to the blood eosinophils of patients in whom systemic steroids were administered only after**

**the blood samples had been taken. Continuous parameters are displayed as Median and (IQR). Chi-Square test was performed to compare gender between the**

**groups. For continuous parameters: Mann-Whitney test was calculated for two groups comparisons, Kruskal-Wallis test with Dunn’s test for three groups**

**comparisons. * significant between <100 and 100-300; # significant between <100 and >300; $ significant between 100-300 and >300; § significant between <2% and**

**≥2%.**

|  | **All** |  | **<100 [eos/µl]** | **100-300 [eos/µl]** | **>300 [eos/µl]** | **p-value** |  | **<2 [%]** | ≥**2 [%]** | **p-value** |
| --- | --- | --- | --- | --- | --- | --- | --- | --- | --- | --- |
| N | 322 |  | 163 | 102 | 57 |  |  | 206 | 116 |  |
| Age | 72.5  (64.0 -83.0) |  | 72.0  (62.0 - 82.0) | 72.0  (65.0 - 81.0) | 78.0  (67.0 - 84.5) | n.s. |  | 72.0  (62.0 - 82.0) | 76.0  (67.0 - 83.0) | n.s. |
| Male sex [%] | 60.6 |  | 54.0 | 70.6 | 61.4 | ^&^ |  | 56.8 | 67.2 | n.s. |
| Packyears | 40.0  (30.0 - 55.5) |  | 40.0  (20.0 - 50.0) | 40.0  (30.0 - 50.00) | 45.0  (30.0 - 60.0) | n.s. |  | 40.0  (25.0 - 50.0) | 40.0  (30.0 - 60.0) | n.s. |
| FEV1[l] | 1.00  (0.73 - 1.41) |  | 0.94  (0.70 - 1.24) | 1.07  (0.73 - 1.49) | 1.13  (0.89 - 1.55) | ^##^ |  | 0.95  (0.69 -1.26) | 1.13  (0.81 - 1.51) | ** |
| FEV1 [%pred.] | 41.3  (30.0 -54.0) |  | 36.8  (28.8 - 51.0) | 43.7  (26.8 - 55.7) | 45.5  (34.8 - 61.5) | ^#^ |  | 37.2  (28.0 - 52.0) | 44.1  (34.0 - 57.5) | * |
| FEV1/VC | 45.2  (36.0 - 55.5) |  | 43.0  (34.5 - 51.8) | 44.4  (35.6 - 58.5) | 49.5  (41.6 - 55.9) | ^#^ |  | 43.0  (34.7 - 52.4) | 47.1  (38.7 - 58.5) | * |

**Supplement table 2 displays the baseline characteristics of patients without any signs of pneumonia (n=322). Continuous parameters are displayed as Median and**

**(IQR). Chi-Square test was performed to compare gender between the groups (& indicates a significantly different gender distribution). For continuous parameters:**

**Mann-Whitney test was calculated for two groups comparisons, Kruskal-Wallis test with Dunn’s test for three groups comparisons. * significant between <100 and**

**100-300; # significant between <100 and >300; $ significant between 100-300 and >300; § significant between <2% and ≥2%.**

|  | **All** |  | **<100 eos/µl** | **100-300 eos/µl** | **>300 eos/µl** | **p-value** |  | **<2%** | **≥2%** | **p-value** |
| --- | --- | --- | --- | --- | --- | --- | --- | --- | --- | --- |
| **N** | 243 |  | 114 | 76 | 53 |  |  | 148 | 95 |  |
| **Leucocytes [g/l]** | 10,5  (8,16 -13,4) |  | 10,6  (8,05 -15,5) | 10,7  (8,37 - 12,5) | 9,98  (7,99 - 13,9) | n.s. |  | 11,2  (8,53 -14,7) | 9,38  (7,66 - 11,8) | ^§§§^ |
| **Neutrophils [%]** | 76,0  (63,0 - 85,3) |  | 83,0  (74,0 - 91,0) | 70,0  (60,0 - 78,0) | 61,0  (54,5 - 74,5) | ^***^  ^###^  ^$^ |  | 82,0  (73,0 - 90,00) | 64,5  (55,8 - 73,5) | ^§§§^ |
| **Procalcitonin [µg/l]** | 0,05  (0,05 - 0,20) |  | 0,10  (0,05 - 0,28) | 0,05  (0,05 - 0,10) | 0,05  (0,05 - 0,05) | ^#^ |  | 0,10  (0,05 - 0,20) | 0,05  (0,05 - 0,05) | ^§^ |
| **Fibrinogen [g/l]** | 5,00  (3,88 - 6,53) |  | 5,14  (4,12 - 6,90) | 5,00  (3,48 - 6,43) | 4,20  (3,50 - 5,30) | ^***^  ^#^ |  | 5,14  (3,90 - 14,0) | 4,60  (3,50 - 5,90) | ^§^ |
| **CRP**  **(mg/l)** | 16,0  (2,60 - 59,0) |  | 25,0  (11,0 - 80,8) | 9,00  (2,50 - 45,3) | 7,55  (2,50 - 24,3) | ^***^  ^###^ |  | 24,0  (8,75 - 74,5) | 7,55  (2,50 - 26,0) | ^§§§^ |

**Supplement Table 3 displays inflammatory markers according to the blood eosinophils of patients in whom systemic steroids were administered only after the**

**blood samples had been taken. Continuous parameters are displayed as Median and (IQR). N=243 for leukocytes, n=238 for neutrophils, n= 86 for Procalcitonin,**

**n=234 for fibrinogen. n=239 for CRP; * significant between <100 and 100-300; # significant between <100 and >300; $ significant between 100-300 and >300;**

**§ significant between <2% and ≥2%.**

|  | **All** |  | **<100 eos/µl** | **100-300 eos/µl** | **>300 eos/µl** | **p-value** |  | **<2%** | **≥2%** | **p-value** |
| --- | --- | --- | --- | --- | --- | --- | --- | --- | --- | --- |
| **N** | 322 |  | 163 | 102 | 57 |  |  | 206 | 116 |  |
| **Leucocytes [g/l]** | 10,9  (8,16 - 14,4) |  | 11,3  (8,16 - 15,5) | 11,2  (8,33 - 13,3) | 9,70  (7,79 - 14,5) | n.s. |  | 12,1  (8,67 - 15,3) | 9,37  (7,67 - 11,9) | ^§§§^ |
| **Neutrophils [%]** | 76,0  (66,8 - 86,0) |  | 82,0  (75,0 - 90,0) | 73,0  (64,0 - 79,3) | 63,0  (55,5 - 76,0) | ^*** ### $$^ |  | 82,0  (74,0 - 89,0) | 66,5  (57,0 - 75,8) | ^§§§^ |
| **Procalcitonin [µg/l]** | 0,05  (0,05 - 0,20) |  | 0,10  (0,05 - 0,20) | 0,10  (0,05 - 0,20) | 0,05  (0,05 - 0,05) | ^#^ |  | 0,05  (0,05 - 0,20) | 0,05  (0,05 - 0,15) | n.s. |
| **Fibrinogen [g/l]** | 4,80  (3,88 - 6,33) |  | 4,90  (4,00 - 6,50) | 4,95  (3,65 - 6,48) | 4,20  (3,50 - 5,40) | n.s. |  | 4,90  (3,90 - 6,65) | 4,55  (3,65 - 5,98) | n.s. |
| **CRP**  **(mg/l)** | 16,0  (4,95 - 60,3) |  | 25,0  (10,0 - 67,5) | 13,0  (2,50 - 47,0) | 7,10  (2,50 - 32,0) | ^** ###^ |  | 22,7  (8,00 - 65,9) | 9,00  (2,50 - 42,2) | ^§§§^ |

**Supplement table 4 displays the subgroup analysis of inflammatory markers according to the blood eosinophils in cases of patients without any signs of pneumonia (n=322). Continuous parameters are displayed as Median and (IQR). * significant between <100 and 100-300; # significant between <100 and >300; $ significant between 100-300 and >300; § significant**

**between <2% and ≥2%.**

| **ICS preadmission** |  | **All** | |  | **<100 eos/µl** | | **100-300 eos/µl** | | **>300 eos/µl** | | **p-value** |  | **<2%** | | **≥2%** | | **p-value** |
| --- | --- | --- | --- | --- | --- | --- | --- | --- | --- | --- | --- | --- | --- | --- | --- | --- | --- |
|  |  | n | % |  | n | % | n | % | n | % |  |  | n | % | n | % |  |
| **No ICS** |  | 175 | 42 |  | 88 | 40.2 | 55 | 42.3 | 32 | 47.1 | n.s. |  | 109 | 39.5 | 66 | 46.8 | n.s. |
| **Beclomethasone** |  | 43 | 10.3 |  | 27 | 12.3 | 10 | 7.7 | 6 | 8.8 | n.s. |  | 31 | 11.2 | 12 | 8.5 | n.s. |
| **Budesonide** |  | 131 | 31.4 |  | 72 | 32.9 | 41 | 31.5 | 18 | 26.5 | n.s. |  | 91 | 33 | 40 | 28.4 | n.s. |
| **Fluticasone propionate** |  | 63 | 15.1 |  | 29 | 13.2 | 23 | 17.7 | 11 | 16.2 | n.s. |  | 41 | 14.9 | 22 | 15.6 | n.s. |

**Supplement table 5 displays the distribution of different types of ICS prescribed according to the eosinophil and non-eosinophil thresholds in cases of patients with a differential cell blood count at the day of admission (n=417). Statistical analysis was performed using Chi-square test. Types of ICS that were administered in less than five cases were excluded from this analysis (Ciclesonide and Fluticasone furoate).**
